# Efficient Generation of Transgenic Buffalos (Bubalus bubalis) by Nuclear Transfer of Fetal Fibroblasts Expressing Enhanced Green Fluorescent Protein

**DOI:** 10.1038/s41598-018-25120-5

**Published:** 2018-05-03

**Authors:** Fenghua Lu, Chan Luo, Nan Li, Qingyou Liu, Yingming Wei, Haiying Deng, Xiaoli Wang, Xiangping Li, Jianrong Jiang, Yanfei Deng, Deshun Shi

**Affiliations:** 1 Guangxi High Education Key Laboratory for Animal Reproduction and Biotechnology, State Key Laboratory for Conservation and Utilization of Subtropical Agro-Bioresources, Guangxi University, Nanning, 530005 China; 2 Reproductive Center of Liuzhou Municipal Maternity and Child Healthcare Hospital, Liuzhou, 545001 China

## Abstract

The possibility of producing transgenic cloned buffalos by nuclear transfer of fetal fibroblasts expressing enhanced green fluorescent protein (EGFP) was explored in this study. When buffalo fetal fibroblasts (BFFs) isolated from a male buffalo fetus were transfected with pEGFP-N1 (*EGFP* is driven by CMV and *Neo* is driven by SV-40) by means of electroporation, Lipofectamine-LTX and X-tremeGENE, the transfection efficiency of electroporation (35.5%) was higher than Lipofectamine-LTX (11.7%) and X-tremeGENE (25.4%, *P* < 0.05). When BFFs were transfected by means of electroporation, more embryos from BFFs transfected with pEGFP-IRES-Neo (*EGFP* and *Neo* are driven by promoter of human elongation factor) cleaved and developed to blastocysts (21.6%) compared to BFFs transfected with pEGFP-N1 (16.4%, *P* < 0.05). A total of 72 blastocysts were transferred into 36 recipients and six recipients became pregnant. In the end of gestation, the pregnant recipients delivered six healthy calves and one stillborn calf. These calves were confirmed to be derived from the transgenic cells by Southern blot and microsatellite analysis. These results indicate that electroporation is more efficient than lipofection in transfecting exogenous DNA into BFFs and transgenic buffalos can be produced effectively by nuclear transfer of BFFs transfected with pEGFP-IRES-Neo.

## Introduction

The production of transgenic animals has numerous potential applications in establishing human genetic disease models, producing phamaceutical proteins through animal mammary gland bioreactors, and improving the growth performance and disease resistance of farm animals^1^. The first transgenic animal was produced successfully in mouse based on the microinjection of foreign DNA into zygotic pronuclei in 1982^2^, and this method was also used to produce the first transgenic livestock in 1985^3^. However, this procedure is characterized by low efficiency (1–5% transgenic offspring), random integration and variable expression of the transgene as well as a considerable proportion of mosaicism^4^. Thus, several alternatives to pronuclear DNA injection have been developed in the last years to improve the efficiency and to reduce the cost of generating transgenic livestock. These include sperm mediated DNA transfer^5^, intracytoplasmic injection (ICSI) of sperm heads carrying foreign DNA^6,7^, injection or infection of oocytes and/or embryos by retro- and lentiviral vectors^8–10^ and the use of somatic cell nuclear transfer (SCNT)^11–15^. Of these alternatives to pronuclear DNA injection, SCNT is the most powerful strategy for generating locus-specific modification of large animals. The genetic modification of donor cells can be completed by cellular transfection and selection, and then the locus-specific modification of animals can be obtained by nuclear transfer of these donor cells^16–18^. Although SCNT offers a new cell-based route for introducing precise genetic modifications in a range of animal species such as cattle^19^, sheep^11^, goats^20,21^ and pigs^22–24^, the production of transgenic animals by SCNT is inefficient and only1–5% of bovine transgenic SCNT embryos developed to term and resulted in live births^25^.

Establishment of viable transgenic cell lines is one of the important steps in the generation of transgenic cloned animals. Numerous chemical and physical approaches have been used to introduce the foreign DNA into mammalian cells including calcium phosphate precipitation, electroporation, lipofection, and viral vectors, but their efficiency is variable and dependent on the cell types to be transfected^26^. Studies related to the transfection of buffalo somatic cells are rarely reported. Thus, establishment of an efficient protocol for introducing foreign DNA into BFFs is essential for generation of transgenic cloned buffalos.

In order to improve the efficiency of producing transgenic cloned animals, enhanced green fluorescent protein (EGFP) that acts as a marker aiding the identification of transgenic animals had been widely employed in the production of transgenic cloned offspring in mice^27^, pigs^22^, goats^28^ and cattle^19^. However, it does not improve the efficiency of producing transgenic animals as the physiological state of donor cells may be altered by G418 that is frequently used for the selection of positive transgenic cells. A long period of selecting cells with G418 causes alteration in cell potentiality and cytological aging^29^. In addition, a bystander effect has been reported in which non-transgenic cells were protected by the secretion of the antibiotic-resistance gene in transgenic cells or by direct cell-to-cell contact^30–32^. As the result of this bystander effect, many presumptive transgenic colonies are polyclonal. Thus, how to obtain healthy transgenic cells are need to be investigated further in future.

Buffalo (Bubalus bubalis) is an important domestic animal distributed in the tropical and subtropical region and is characterised by high fat and protein content in milk. These characters will confer buffalo as a good bioreactor candidate to produce phamaceutical protein through introducing medicinal value genes. We have previously demonstrated the production of cloned buffalos by SCNT^33^. However this employed non-genetically modified donor cells and the generation of transgenic cloned buffalos by SCNT using transgenic donor cells has not yet been reported. Thus, the present study was undertaken to extend our works by investigating the effects of transfection method and vector structure on the efficiency of producing transgenic BFF, and then explore the possibility of producing transgenic cloned buffalos by SCNT.

## Results

### Transfection efficiency of electroporation and cationic lipids

**Figure 1:**
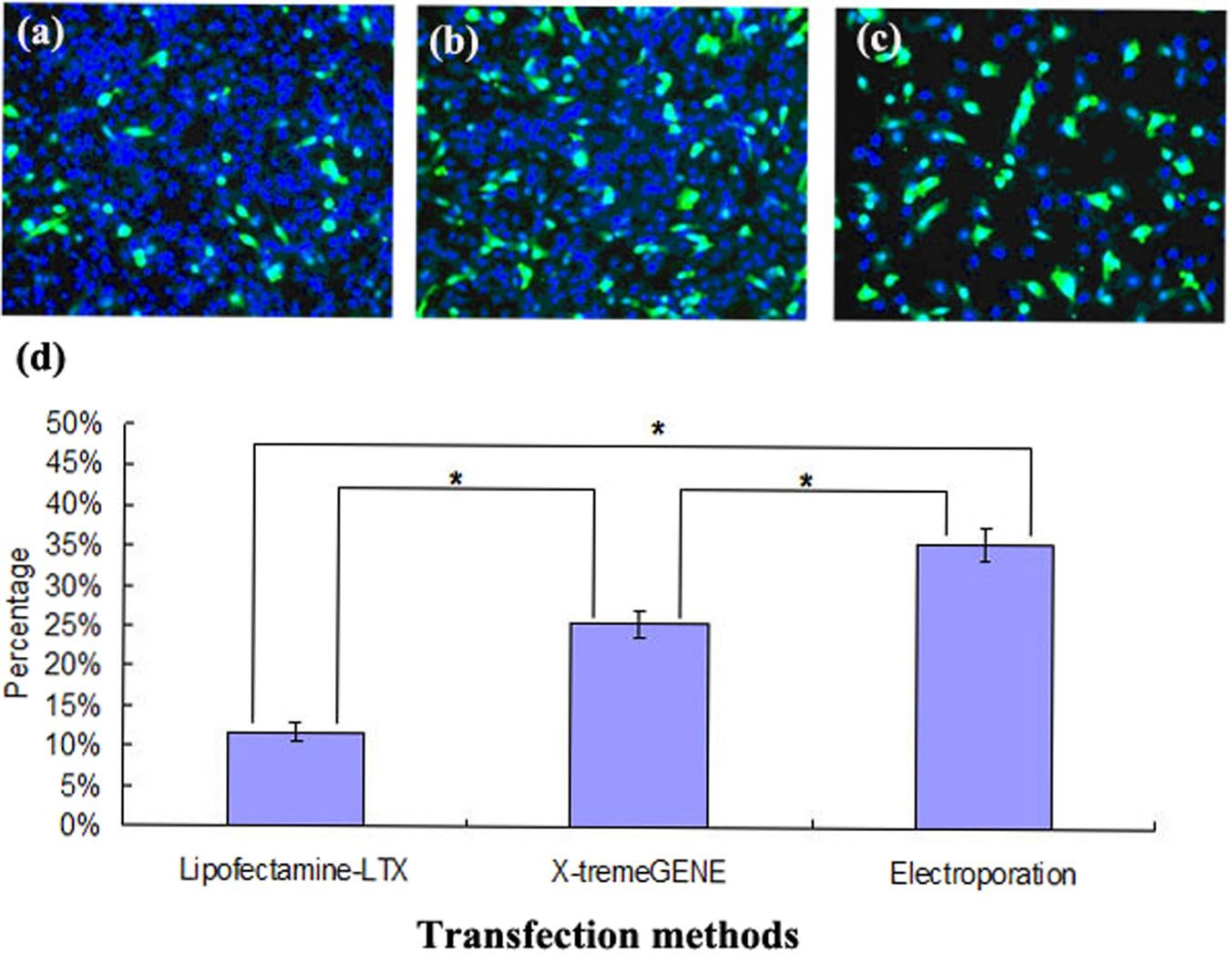
BFFs transfected with pEGFP-N1 by means of lipofectamine-LTX (**a**), X-tremeGENE (**b**), electroporation (**c**) and their percentage expressing EGFP (**d**). Nuclei were stained with Hoechst33342. Data are from three replicates (n = 3), presented as mean ± SEM and analyzed by one-way ANOVA (*P < 0.05).

To determine the optimal method for transfection of plasmid DNA into buffalo fetal fibroblasts (BFFs), the transfection efficiency of electroporation, Lipofectamine-LTX and X-tremeGENE were compared (Fig. 1). More BFFs transfected by means of electroporation expressed EGFP (35.5 ± 4.5%) in comparison with BFFs transfected by means of Lipofectamine-LTX (11.7 ± 3.1%) and X-tremeGENE (25.4 ± 4.5%, *P* < 0.05). The transfection efficiency of X-tremeGENE was also higher than Lipofectamine-LTX (*P* < 0.05).

### Transgenic efficiency of different EGFP vectors

To examine the influence of vector structure on the transfection efficiency of BFFs and their subsequent embryonic development after nuclear transfer, BFFs were transfected with the linearized DNA fragments of pEGFP-IRES-Neo or pEGFP-N1 by means of electroporation, and then cultured in the medium containing 600 μg/mL of G418 for 7–14 days at 24 h after transfection to select EGFP positive colonies (Supplementary Information Figure S1). The EGFP-positive colonies appeared earlier in BFFs transfected with pEGFP-IRES-Neo compared to pEGFP-N1. Significantly more EGFP positive colonies formed from BFFs transfected with pEGFP-IRES-Neo in comparison with pEGFP-N1 (Supplementary Information Table S1, P < 0.05). When these transgenic cells were transferred into enucleated oocytes (Table 1), more embryos reconstructed with BFFs that had been transfected with pEGFP-IRES-Neo cleaved (73.22%) and developed to blastocysts (21.55%) in comparison with the BFFs transfected with pEGFP-N1 (57.77% and 16.39%, *P* < 0.05). However, the blastocyst yield of SCNT embryos from transgenic BFFs was lower than that of non-transgenic BFFs that were isolated from the same fetus, stored in liquid nitrogen and passaged 3 to 5 times after thawing (28.2%, *P* < 0.05).

**Table 1:** *In vitro* development of SCNT embryos derived from BFFs transfected with different vectors.

Donor Cells	NT Embryos	Cleaved (%)	Blastocysts developed (%)
BFF trasfected with pEGFP- N1	421	243 (57.77)^b^	69 (16.39)^c^
BFF transfected with pEGFP-IRES-Neo	362	265 (73.2)^a^	78 (21.55)^b^
BFF without transfection	312	241 (77.2)^a^	88 (28.2)^a^

Data were from more than three replicates.

^a,b^Within a column, values with different superscripts are significantly different (*P* < 0.05).

### Production of transgenic cloned buffalos

**Figure 2:**
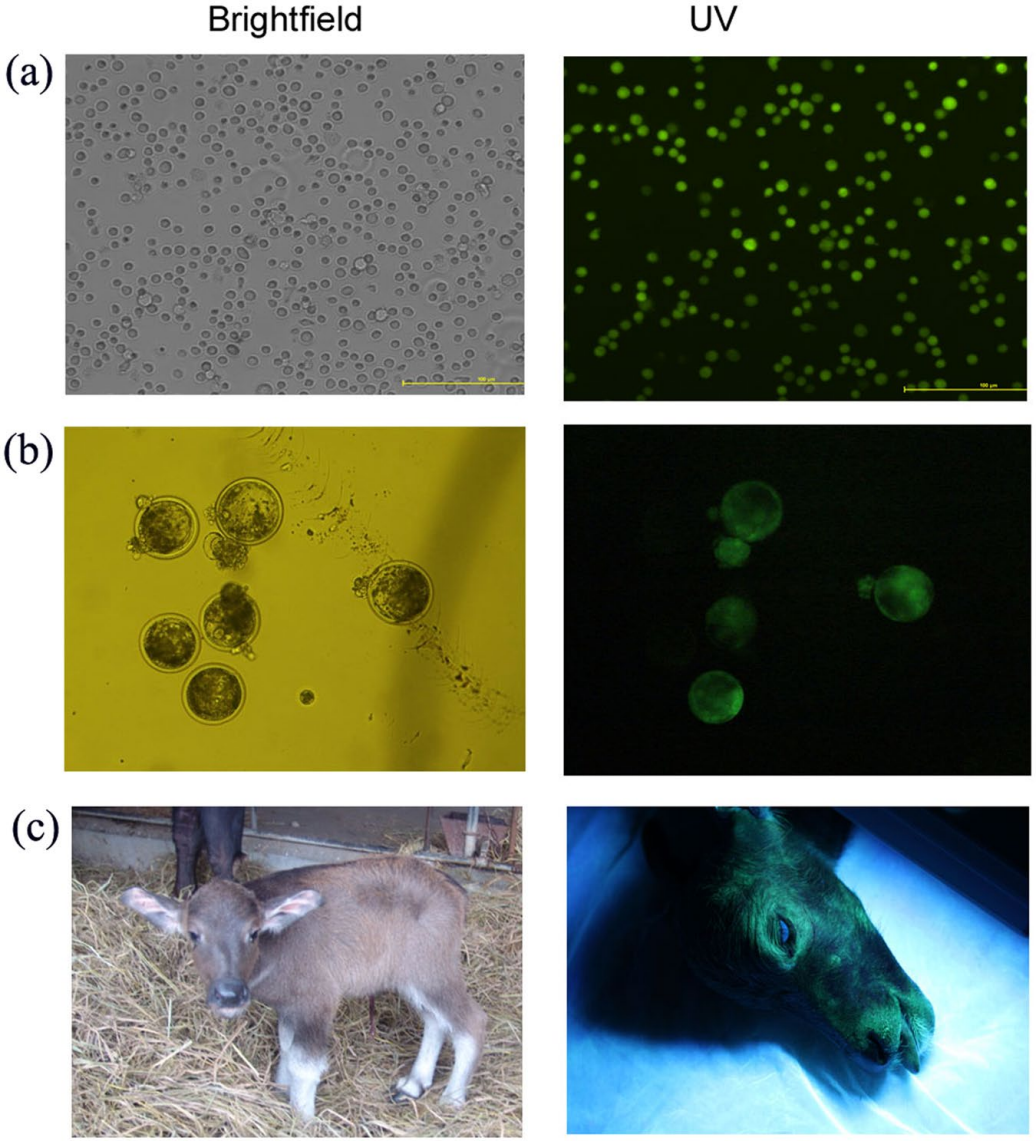
Expression of EGFP in transgenic BFFs (**a**, 200×), SCNT blastocysts (**b**, 100×) and SCNT calf (**c**).

According to the results of above experiments in which electroporation was found to be more efficient than lipofection in transfecting plasmid DNA into BFFs and more SCNT embryos from BFFs transfected with pEGFP-IRES-Neo cleaved and developed to blastocysts compared to pEGFP-N1, electroporation and pEGFP-IRES-Neo were employed for production of transgenic buffalo SCNT embryos. BFFs were transfected with linearized DNA of pEGFP-IRES-Neo by means of electroporation, selected by 14 days of G418 culture and identified by PCR. Then, transgenic BFFs with normal morphology (Fig. 2a) were selected for producing transgenic SCNT embryos (Fig. 2b). A total of 72 blastocysts expressing EGFP were transferred non-surgically into the uterine horn of 36 recipient buffalos at Day 6 of the native estrous cycle, and 12 recipients were confirmed to become pregnant by rectal palpation at Day 60 of gestation. Finally, six recipients maintained pregnancy to term, five of them delivered five healthy male calves, one recipient delivered one healthy calf and one stillborn calf (Table 2, Fig. 2c).

**Table 2:** Pregnancy and calf birth following transfer of transgenic SCNT embryos to recipient buffalos.

Recipients	Embryos transferred	No. pregnant recipients at D60 (%)	Calves born	No. embryos developed to term (%)
36	72	12 (33.3)	6 live calves, 1 stillborn calf	7/72 (9.7)

### Analysis of EGFP expression in cloned offspring

**Figure 3:**
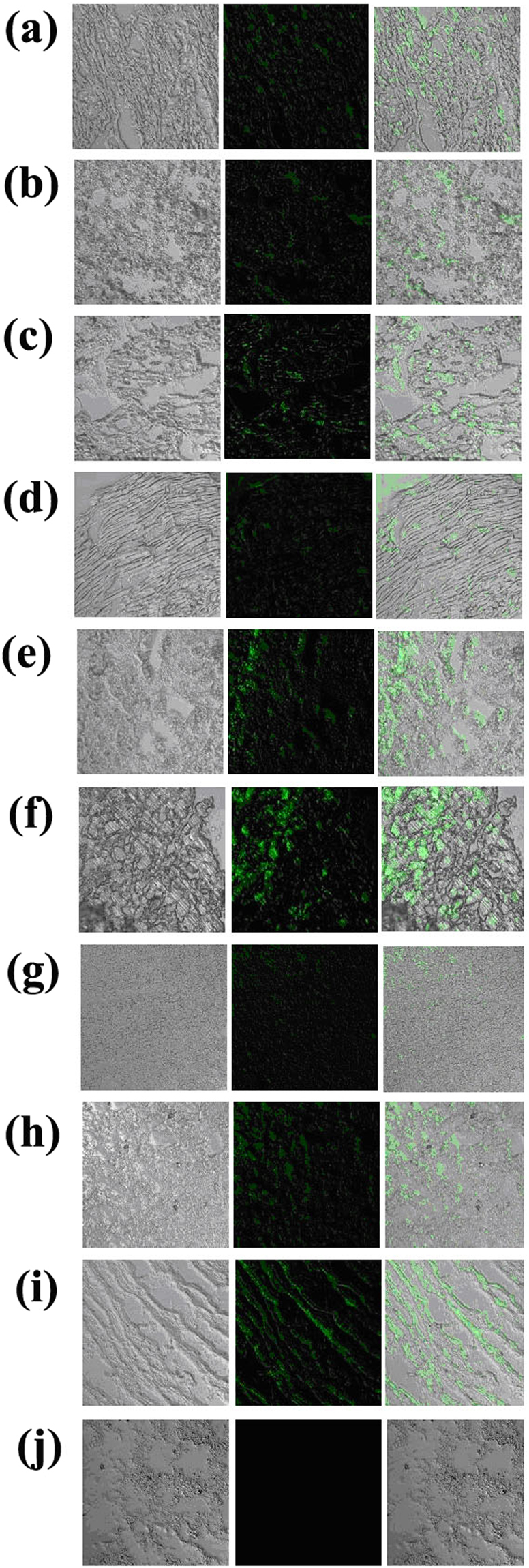
Expression of EGFP in tissues (×200) from transgenic cloned calf: heart (**a**), lung (**b**), muscle (**c**), diaphragm (**d**), testis (**e**), fat (**f**), spleen (**g**), kidney (**h**), intestines (**i**) and non-transgenic calf: lung (**j**).

As showed in Fig. 2c, the expression of EGFP was observed in the head of the new-born calf under the UV light. Moreover, the expression of EGFP in tissues (Fig. 3) and ear skin primary fibroblast cells (Supplementary Information Figure S2) from the stillborn cloned calf was also detected under the laser scanning confocal microscope (LSCM). However, the expression levels of EGFP varied in different tissues and different cells within the same tissue, as the intensity of green fluorescence was strong in testis, fat, kidney and intestines, relatively weak in muscle, diaphragm, heart, lung and spleen.

### Genetic identification of transgenic cloned buffalos

**Figure 4:**
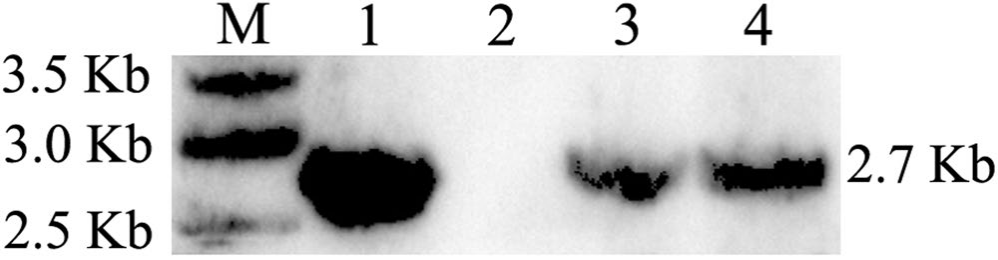
Analysis of transgene integration in the cloned calf by Southern blot. Lane M: molecular size markers (1-kb ladder, Invitrogen); Lane 1: positive control; Lane 2: non-transgenic cells; Lane 3: transgenic cells; Lane 4: cells from the ear tissue of transgenic cloned calf.

To identify whether the cloned buffalo calves were derived from donor cells, a microsatellite analysis of genomic DNA from the various samples were performed using ten microsatellite markers. The comparison of pattern of alleles in cloned buffalo calves with their donor cell populations and surrogate mothers indicated that three healthy transgenic cloned calves (CB1, CB2, CB3) were derived from the transgenic BFFs, as there was 100% identity to donor cells (BFFs integrated with pEGFP-IRES-Neo) and a significant difference from the surrogate mothers (RB1, RB2, and RB3) at all ten microsatellite markers (Table 3). The integration of EGFP and Neo gene in the genomic DNA of stillborn cloned buffalo was also confirmed by Southern blot as the genomic DNA from the ear tissues of transgenic cloned calf could hybridize with EGFP and *Neo* probe, while the genomic DNA from non-transgenic cells could not hybridize with EGFP and *Neo* probe (Fig. 4).

**Table 3:** Microsatellite analysis of buffalos and donor cells^*^.

Allele of mi- crosatellite markers	Buffalos/donor cells						
	CB1	CB2	CB3	RB1	RB2	RB3	Donors
BM1818	268/270	268/270	268/270	264/268	264/270	264/270	268/270
CSSM66	174/178	174/178	174/178	176/178	178/184	176/184	174/178
ILSTS019	174/176	174/176	174/176	176/180	176/180	172/176	174/176
ILSTS020	136/142	136/142	136/142	136/138	138/140	136/138	136/142
ILSTS023	160/161	160/161	160/161	161/162	163/NA	160/161	160/161
ILSTS030	155/157	155/157	155/157	157/NA	157/161	155/157	155/157
ILSTS031	251/255	251/255	251/255	253/255	253/NA	253/257	251/255
ILSTS058	130/136	130/136	130/136	130/136	130/142	142/144	130/136
ILSTS061	140/153	140/153	140/153	149/163	138/149	140/157	140/153
ILSTS086	176/185	176/185	176/185	174/181	178/185	178/182	176/185

^*^Microsatellite analysis was performed on genomic DNA from three lived transgenic cloned buffalo calves (CB1, CB2 CB3), surrogate buffalo cows (RB1, RB2, RB3) and donor cells.

## Discussion

Buffalo is an important domestic animal distributed in the tropical and subtropical regions and characterized with strong disease resistance, excellent tolerance to high temperature and humidity, and high content of fat and protein in milk. These characters will confer buffalo as a good bioreactor candidate to produce phamaceutical protein through introducing medicinal value genes. As a powerful protocol for generating locus-specific modification of animals, although SCNT has been employed successfully for generating transgenic cloned animals such as cattle^19^, sheep^11^, goats^20,21^ and pigs^22–24^, the generation of transgenic cloned buffalos by SCNT has not been reported. The results of our study first demonstrated that transgenic buffalos can be produced effectively by nuclear transfer of transgenic cell lines.

Preparation of transgenic cell lines is the first important step of generating transgenic cloned animals. There are many ways for transfecting exogenous genes into mammalian cells. The advantage of transfection by electroporation is that it is less chemically toxic when compared with other methods^34^, but the transfection efficiency is highly dependent on the cell types. In the present study, electroporation was proved to be more efficient in transfecting plasmid DNA into BFFs compared to lipofection^35^.

The efficiency of transgenic cloning may be related to the physiological state of donor cells. The extended period of selecting culture and transfecting treatment may shorten the normal life span of somatic cells^36^. Thus, an effective protocol for selecting transgenic cells is considerable importance^37^. Previous reports showed that many presumptive transgenic colonies were mixed and contained both transgenic and non-transgenic cells due to the bystander effect of which non-transgenic cells were protected by the antibiotic-resistant secretions of transgenic cells^30–32^. To obtain healthy transgenic cells, expression of the antibiotic-resistance gene (*Neo*) should be regulated to an appropriate level and the selecting period with G418 should be as short as possible. In this work, transgenic colonies were found to form much earlier in the BFFs transfected with pEGFP-IRES-Neo in comparison with pEGFP-N1. It may be due to the relatively lower expression of *Neo* in BFFs transfected with pEGFP-IRES-Neo in comparison with pEGFP-N1. The *Neo* in pEGFP-N1 was driven by its own promoter (SV-40), whereas the *Neo* in pEGFP-IRES-Neo was driven by the constitutive promoter (human elongation factor, EF) of EGFP separated by an upstream internal ribosome entry site (IRES) sequence. It had been reported that IRES-dependent second gene expression is significantly lower than cap-dependent first gene expression^38,39^. The lower expression of *Neo* may result in less bystander effects and non-transgenic cells will be killed by G418 in shorter time. As a result, transgenic cells will grow quickly through escaping from the competition of non-transgenic cells and then form transgenic colonies with good morphology in shorter time. In addition, we also observed that some G418 resistant clones from BFFs transfected with pEGFP-N1 did not express EGFP (unpublished data) due to that the Neo cassette is driven by its own promoter. The pEGFP-IRES-Neo might overcome this problem and guaranteed that G418 resistant clones express EGFP as the *EGFP* and *Neo* are driven by the constitutive promoter. Therefore, regulation of *Neo* expression by optimizing the vector structure and shortening the period of G418 selection could improve the physiological state of donor cells and then increase the efficiency of transgenic cloning.

In the present study, the percentage of SCNT embryos from transgenic BFFs developing to blastocysts was lower than the SCNT embryos from BFFs without transfection, which was similar to the reports in cattle^40,41^. Zakhartchenko *et al*. thought that the decrease in embryonic development of SCNT embryos from transfected cells was due to the extended culture period for transfection and selection^41^. However, there were also some reports indicating that no significant difference was found in the *in vitro* developmental ability of cloned embryos from transfected and non-transfected cells in cattle, goats and pigs^19,20,23^. These discrepancies may be due to the species, vector types, or different SCNT protocols.

The enhanced fluorescent protein (EGFP) is visible under the fluorescent microscope and has been applied widely in cell biological and molecular biological research^42^. Previous report indicated that EGFP may reduce viability of cells transfected with this gene^43^. In this study, the blastocyst yield of SCNT embryos from BFFs transfected with *EGFP* was also lower than BFFs without transfection, but nearly 10% of transgenic cloned blastocysts developed to the term of gestation and delivered cloned calves after transfer to recipients, indicating that transgenic buffalos can be generated efficiently by nuclear transfer of BFFs transfected with *EGFP*.

In the present study, the EGFP expression in one stillborn transgenic cloned calf was found to vary in different tissues and different cells within the same tissue. This result was consistent to the situation of cloned cattle^44^, pigs^45^ and rabbits^46^. The variable expression of transgene in different tissues may be associated with their cellular differentiation state and controlled by the epigenetic modification^47^. Thus, a tissue specific promoter is often employed in the production of transgenic animals according to practical purpose, such as the promoter of beta-casein that is used in mammary gland bioreactor.

In conclusions, exogenous DNA can be transfected into BFFs efficiently by means of electroporation and the structure of vectors has a profound influence on the physiological state of transfected BFFs and their embryonic development after nuclear transfer. Transgenic buffalos can be produced effectively by nuclear transfer of transgenic cell lines.

## Materials and Methods

### Ethics Statements

All animals were handled in strict accordance with good animal practices as defined by the relevant national and/or local animal welfare bodies. All animal experimental protocols were approved by the Animal Care and Use Committee of Guangxi University, China, and performed in accordance with animal welfare and ethics guidelines. All efforts were made to minimize animals’ pain, suffering, and to reduce the number of animals used.

### Reagents and media

All chemicals used were purchased from Sigma Chemical Company (St. Louis, MO, USA), with the exception of TCM 199 powder that was purchased from Gibco BRL (Paisley, Scotland, UK), and fetal bovine serum (FBS) and Dulbecco’s Modified Eagle’s Media (DMEM) that were purchased from Invitrogen Company (Carlsbad, CA, USA). The preparation of media used in this study including *in vitro* maturation (IVM) medium, embryo culture medium (CM) and basic micromanipulation medium was described by Shi *et al*.^33^.

### Structure of transgene cassettes

**Figure 5:**
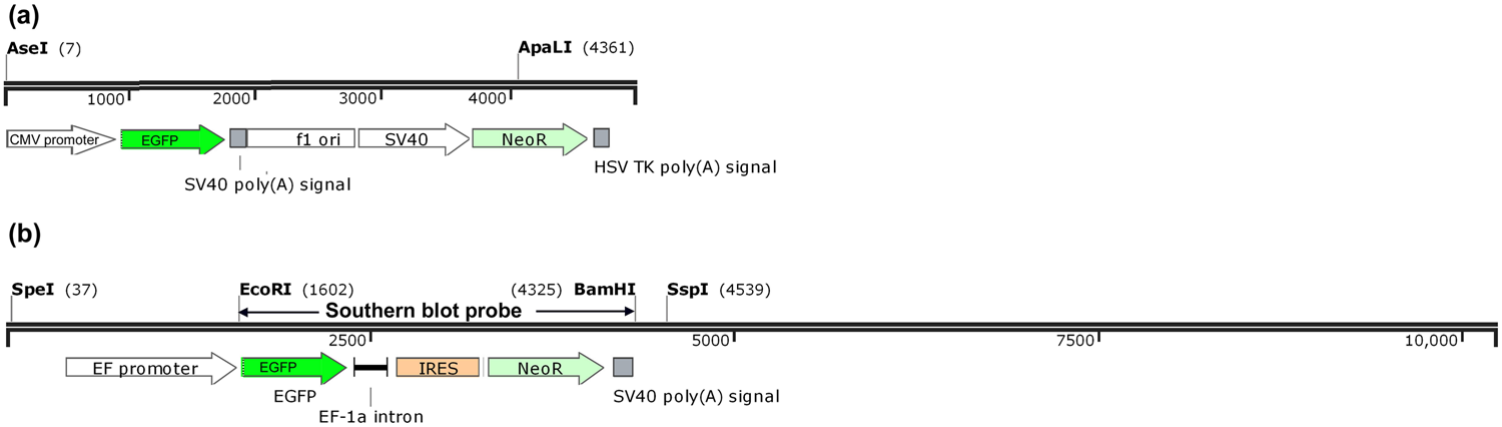
Schematic structure of linearized plasmids. (**a**) Structure of pEGFP-N1. (**b**) Structure of pEGFP-IRES-Neo, approximate positions of the Southern blot probe and restriction enzyme sites are illustrated. The drawing is not to scale.

Two vectors (Fig. 5) were constructed for transfection of BFFs. The pEGFP-N1 vector was purchased from Clontech (MountainView, CA, USA), which was 4.7 kb of length and consisted of an enhanced version of EGFP-reporter gene driven by cytomegalovirus (CMV) promoter and a neomycin-resistance cassette driven by SV-40 promoter (Fig. 5a). The pEGFP-IRES-Neo vector was kindly provided by Prof Ning Li (China Agricultural University, Beijing, China), which was 10.2 kb of length and consisted of an EGFP-reporter gene and a neomycin-resistance cassette driven by the promoter of EF with an upstream IRES sequence of *Neo* (Fig. 5b). To eliminate the effect of DNA fragment size on the transfection efficiency, the redundant backbone of vectors was removed before transfection. The pEGFP-N1 vector was digested by A*sel* I and A*pal* I to generate a 4.3 kb linearized DNA fragment, and the pEGFP-IRES-Neo vector was also digested by S*pe* I and S*sp* I to generate a 4.3 kb linearized DNA fragment.

### Isolation and culture of BFFs

Buffalo fetal fibroblasts were isolated from a male buffalo fetus at approximately 90 days of gestation according to the method previously described^48^. Briefly, tissues from the ear skin of a male buffalo fetus were minced with sterile scissors in a 35 mm Petri dish (Nunc, Roskilde, Denmark), then cultured in high glucose-DMEM supplemented with 100 U/ml Penicillin, 0.1 mg/ml Streptomycin and 10% FBS under a humidified 5% CO_2_ in air at 37.5 °C. After 7 to 10 days of culture, confluent fibroblast monolayers were obtained and then routinely passaged using an enzymatic solution (0.25% trypsin and 0.05% EDTA) for 7 min. Small aliquots of progressively growing cell lines that were established were frozen in DMEM supplemented with 10% FBS and 10% dimethyl sulfoxide (DMSO) for further study.

### Transfection and selection of transgenic cells

BFFs stored in liquid nitrogen were thawed 1–2 days prior to transfection and cultured to approximately 80% of confluence on the day of experiment. Cells were trypsinized with a solution of 0.25% trypsin and 0.05% EDTA, resuspended in electroporation buffer to give a density of 5 × 10^6^ cells/mL and transferred to an electroporation cuvette (4‐mm gap, BTX). Then, cells were transfected with 30 μg linearized plasmid DNA by means of electroporation (a 300 V/cm pulse for 10 msec) using an ECM2001 Electrocell Manipulator (BTX Inc., San Diego, CA). Following electroporation, the cells were resuspended in cell culture medium and cultured in a 6‐well dish under humidified 5% CO_2_ in air at 37.5 °C. For transfection with cationic lipids, BFFs at 80% of confluence were transfected with 30 μg linearized plasmid DNA by means of Lipofectamine-LTX (Invitrogen, CA, USA) or X-tremeGENE (Roche, Basel, CH) according to the manufacturer’s instructions.

To determine the transfection efficiency, cells were fixed for 10 min with 4% freshly prepared paraformaldehyde in PBS at 24 h after transfection at room temperature, and stained with 10 μg/mL Hoechst 33342 (H33342). The percentage of cells expressing EGFP (green and blue) was determined under an inverted fluorescence microscope (5 fields per dish). The transfection efficiency was defined as the percentage of EGFP positive cells with at least of 500 cells.

For selection of transgenic cells, at 24 h after transfection with the efficient method that had been proved, the culture medium was replaced with the selection medium containing 600 ng/mL G418 and put into culture for 14 days. The selection medium was replaced with fresh medium every 48 h, and then EGFP-positive and G418-resistant colonies were picked up in the end of selection. Finally, EGFP-positive and G418-resistant cells were expanded and frozen in liquid nitrogen for subsequent SCNT.

### *In vitro* maturation of oocytes

Chinese swamp buffalo ovaries were obtained from a local abattoir. Ovaries were excised within 20 to 30 min after slaughter and were transported to the laboratory within 4 h in a thermos containing PBS at 35 to 37 °C. Buffalo cumulus-oocyte complexes (COCs) were recovered by aspiration of follicles in diameter of 2 to 6 mm using a 10 mL disposable syringe with an 18-gauge needle. COCs with multi-layers of cumulus cells were selected for IVM. Then, COCs were washed twice in the IVM medium (TCM-199 supplemented with 26.2 mM NaHCO_3_, 5 mM HEPES, 5% OCS, 2% bovine follicular fluid and 0.1 µg/mL FSH) and cultured in a 30 mm glass dish containing 1.5 mL IVM medium under a humidified atmosphere of 5% CO_2_ in air at 38.5 °C.

### Somatic cell nuclear transfer

Somatic cell nuclear transfer was carried out as described previously^33^. Briefly, after IVM for 22 h, surrounding cumulus cells of oocytes were removed by manual pipetting in CM containing 2 mg/mL hyaluronidase, and then oocytes with an extruded first polar body were selected for enucleation. Selected oocytes were placed into the manipulation medium drop supplemented with 5 μg/mL Cytochalasin B and 0.1 M sucrose for 10 min before micromanipulation. The first polar body and metaphase-II plate were removed by aspiration with a 25 μm inner diameter beveled pipette under a Nikon TE300 inverted microscope equipped with a Narishige micromanipulator (Tokyo, Japan) and Spindle View system (Woburn, MA, USA). Then, trypsinized EGFP-positive cells were transferred into the perivitelline space of enucleated oocytes under an inverted microscope equipped with a G-2A filter (EX: 510–560 nm, BA: 590 nm). The couplet was transferred to a droplet of 100 µL fusion medium (0.28 M mannitol, 0.1 mM CaCl_2_, 0.1 mM MgSO_4_, 5 mM Hepes and 0.1% BSA) overlaid with mineral oil, and then placed on the micromanipulator with two platinum needle electrodes (0.2 mm apart). The fusion was induced by application of an AC pulse of 2 V for 1 sec, followed by three DC pulses of 1 kV/cm for 15 µs using an ECM 2001 Electrocell Manipulator (BTX Inc.,San Diego, CA, USA). Couplets were then washed in CM and incubated in this medium for 30 min at 38.5 °C. The fusion of couplets was checked at ×200 magnification under an inverted microscope. At 2 hours after fusion, activation of fused embryos was induced by exposure to 5 µM Ionomycin in CM for 5 min and subsequent incubation in 2 mM 6-dimethylamino-purine for 3 h at 38.5 °C and 5% CO_2_ in air.

### *In vitro* culture of NT embryos

After activation, reconstructed embryos were placed into co-culture with granulosa cell monolayers in a 30 µL droplet of CM overlaid with mineral oil under a humidified atmosphere of 5% CO_2_ in air at 38.5 °C. Granulosa cell monolayers were established 48–72 h before introduction of embryos. After introduction of embryos, half of the medium was replaced with fresh medium every 48 h. After two days of co-culture, cleavage of reconstructed embryos was checked, and the number of developed blastocysts was recorded within eight days of co-culture.

### Estrous synchronization of recipients and embryo transfer

Estrous synchronization of recipients was induced by injecting 500 mg of the prostaglandin-F2α analogue cloprostenol (Estrumate; Sch-ering Canada, Inc., Montreal, PQ, Canada). Blastocysts that had been confirmed to express EGFP were transferred non-surgically into the uterine horn ipsilateral to the ovary containing a palpable corpus luteum of recipient buffalos at day 6 of estrous cycle. Each recipient received two embryos and the pregnancy status was determined on 60 days after embryo transfer by rectal palpation.

### Analysis of EGFP expression in transgenic cloned calf

Cells isolated from the ear skin of transgenic cloned calves were placed on the inverted fluorescence microscope for analyzing the expression of EGFP. Meanwhile, tissues from brain, heart, lung, liver, kidney, pancreas, spleen, bladder, intestine, testis and muscle of transgenic cloned calves were snap-frozen at −80 °C, cryo-sectioned and mounted onto slides for analyzing the expression of EGFP under a laser scanning confocal microscope (LSCM, Carl Zeiss, Inc., Germany).

### Microsatellite analysis of transgenic cloned calves

To identify whether the transgenic cloned buffalo calves derived from donor cells, a microsatellite analysis of genomic DNA were performed using ten microsatellite markers (BM1818, CSSM66, ILSTS019, ILSTS020, ILSTS023, ILSTS030, ILSTS031, ILSTS058, ILSTS061, andILSTS086). The total genomic DNA was extracted from the ear skin of three newborn calves (CB1, CB2, and CB3) and three foster mothers (RB1, RB2 and RB3). The DNA of donor cells was prepared from transgenic BFFs frozen in liquid nitrogen. The PCR primers for microsatellite markers labeled with fluorescent dyes (6FAM, 9 HEX, and 5 TET) were synthesized by Beijing AUGCT Biotechnology Co. Ltd. The PCR analysis was carried out for 35 cycles, and products were separated by 2% agarose gels. All multiplexing and loci evaluations were performed on an ABI 3730XL automated sequencer by GeneMapper v. 3.5 software (Applied Biosystems, CA, USA).

### Analysis of transgene integration by Southern blot

Genomic DNA was extracted and purified from tissues of transgenic calf using a Wizard^®^Genomic DNA Purification Kit (Promega, WI, USA). Then, genomic DNA (20 mg) were digested with *Eco*R I and *Bam* HI (Promega, WI, USA), separated by electrophoresis in 1.0% agarose gel and transferred to a N+ nylon membrane (Amersham). A 2.7-kb probe (50 ng) from *EGFP* and *Neo* gene-coding sequence was prepared by digesting the pEGFP-IRES-Neo vector with *Eco*R I and *Bam*H I. Probes were labeled with α^32^P dCTP (3000 Ci/mol) using a random primer DNA labeling kit (Pharmacia Biotech) according to the manufacturer’s instructions. Hybridization was carried out following standard procedures.

### Statistical analysis

Within each experiment, the difference between treatments in frequencies of oocytes undergoing cleavage and developing to the blastocyst stage was analyzed by the chi-square test. The data of cell transfection efficiency was expressed as mean ± standard error with at least three replicates for each experiment. Statistical analysis was performed with one‐way ANOVA among groups using SPSS 17.0 (SPSS Inc., Chicago, IL, USA). Probability values less than 0.05 were considered to be statistically significant.

## Electronic supplementary material

Supplementary Information
