# Supplementary material for: Efficient Generation of Transgenic Buffalos (Bubalus bubalis) by Nuclear Transfer of Fetal Fibroblasts Expressing Enhanced Green Fluorescent Protein
Source: Sci Rep. 2018 May 3;8:6967. doi: 10.1038/s41598-018-25120-5 (PMC5934360; doi:10.1038/s41598-018-25120-5)

Supplementary Information of Efficient Generation of Transgenic Buffalos (Bubalus bubalis) by Nuclear Transfer of Fetal Fibroblasts Expressing Enhanced Green Fluorescent Protein

Fenghua Lu[1, +], Chan Luo[1, +], Nan Li[1, 2, +], Qingyou Liu[1], Yingming Wei[1], Haiying Deng[1], Xiaoli Wang[1], Xiangping Li[1], Jianrong Jiang[1], Yanfei Deng[1] &Deshun Shi[1,*]

Table S1. The effect of vector structure on the formation of transgenic colonies

Vectors	No. of original transfected cells	No. of EGFP positive colonies	Transfected efficiency
pEGFP- N1	$4×10^6$	12	$3.00×10^{-6}$（$12/4×10^6$）[a]
pEGFP-IRES-neo	$4×10^6$	122	$3.05×10^{-4}$（$122/4×10^6$）[b]

Data presented were from more than three replicates.

[a-b] Within a column, values with different superscripts are significantly different（$P < 0.05$）.

Figure S1

The G418 resistant colony expressed EGFP. (a) a single G418 resistant colony formatted after 14 days selection (40×), (b) EGFP expression in the colony, observed by fluorescence microscope (40×).

(a) (b)

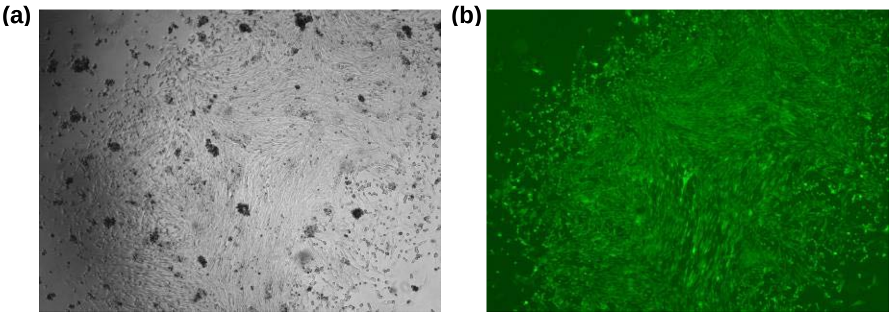

Figure S2

EGFP expression in primary fibroblast cell line established from ear biopsy specimens obtained from transgenic cloned buffalo.

Brightfield Darkfield

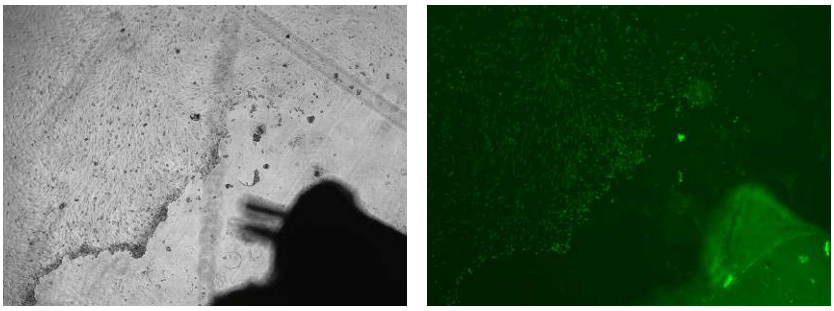

Supplement: Supplementary file 1 — Supplementary Information [file 41598_2018_25120_MOESM1_ESM.pdf]
